# Application of calcium overload-based ion interference therapy in tumor treatment: strategies, outcomes, and prospects

**DOI:** 10.3389/fphar.2024.1352377

**Published:** 2024-02-15

**Authors:** Shuangjiang Li, Ruicheng Fan, Yuekai Wang, Kunqian He, Jinhe Xu, Hongli Li

**Affiliations:** ^1^ Chongqing Key Laboratory of Neurobiology, Department of Teaching Experiment Center, College of Basic Medicine, Army Medical University, Chongqing, China; ^2^ Battalion, College of Basic Medicine, Army Medical University, Chongqing, China

**Keywords:** calcium overload, ion interference therapy, outcome, prospect, strategy, tumor treatment

## Abstract

Low selectivity and tumor drug resistance are the main hinderances to conventional radiotherapy and chemotherapy against tumor. Ion interference therapy is an innovative anti-tumor strategy that has been recently reported to induce metabolic disorders and inhibit proliferation of tumor cells by reordering bioactive ions within the tumor cells. Calcium cation (Ca^2+^) are indispensable for all physiological activities of cells. In particular, calcium overload, characterized by the abnormal intracellular Ca^2+^ accumulation, causes irreversible cell death. Consequently, calcium overload-based ion interference therapy has the potential to overcome resistance to traditional tumor treatment strategies and holds promise for clinical application. In this review, we 1) Summed up the current strategies employed in this therapy; 2) Described the outcome of tumor cell death resulting from this therapy; 3) Discussed its potential application in synergistic therapy with immunotherapy.

## 1 Introduction

Toxicity to normal tissues and drug resistance have long limited the application of traditional tumor therapies, such as radiotherapy, chemotherapy, and immunotherapy. Even worse, there continues to be a rapid increase in the number of tumor patients worldwide. According to the “*Global Cancer Report 2020*” issued by the World Health Organization (WHO), the estimated number of new cancer cases worldwide in 2020 is approximately 19.3 million, with around 10 million cancer-related deaths (Sung et al., 2021). The global burden of cancer is estimated to reach 28.4 million cases by 2040, an increase of 47% compared to 2020. Therefore, it is urgent to develop novel cancer therapies to alleviate patient pain and improve patient outcomes.

Ca^2+^, an essential intracellular second messenger, plays an irreplaceable role in different kinds of fundamental physiological processes including cell cycle, gene expression, intracellular transport and cell death (Giorgi et al., 2018). Considering the significance of Ca^2+^, there are various calcium regulatory mechanisms, including transmembrane transport, mitochondrial and endoplasmic reticulum (ER) buffering, maintaining the concentration and distribution of intracellular Ca^2+^ at a physiological level. Nevertheless, the artificial manipulation of calcium regulatory mechanisms can disturb intracellular calcium homeostasis and potentially induce calcium overload. Calcium overload could cause severe affects to cell function, which mediates irreversible damage and even death of cells. Recently, researchers have reported a strategy that might potentially lead to a novel anti-tumor therapy, called calcium overload-based ion interference therapy (Zhang et al., 2019; Bai et al., 2022).

Calcium overload is a prevalent pathophysiological mechanisms of cell death, resulting in obvious alterations in cell structure and function due to the abnormal accumulation of Ca^2+^(Petersen et al., 2021). A growing number of evidence suggests that calcium overload has an irreplaceable role in tumor therapy. Although modestly increased Ca^2+^ have been reported to promote tumor proliferation and metastasis (Cui et al., 2017), calcium overload could reverse the pro-proliferative effect to a pro-apoptotic effect, which significantly inhibits tumor proliferation and improves drug susceptibility (Peters et al., 2017; Wang et al., 2019; Patergnani et al., 2020).

**FIGURE 1:**
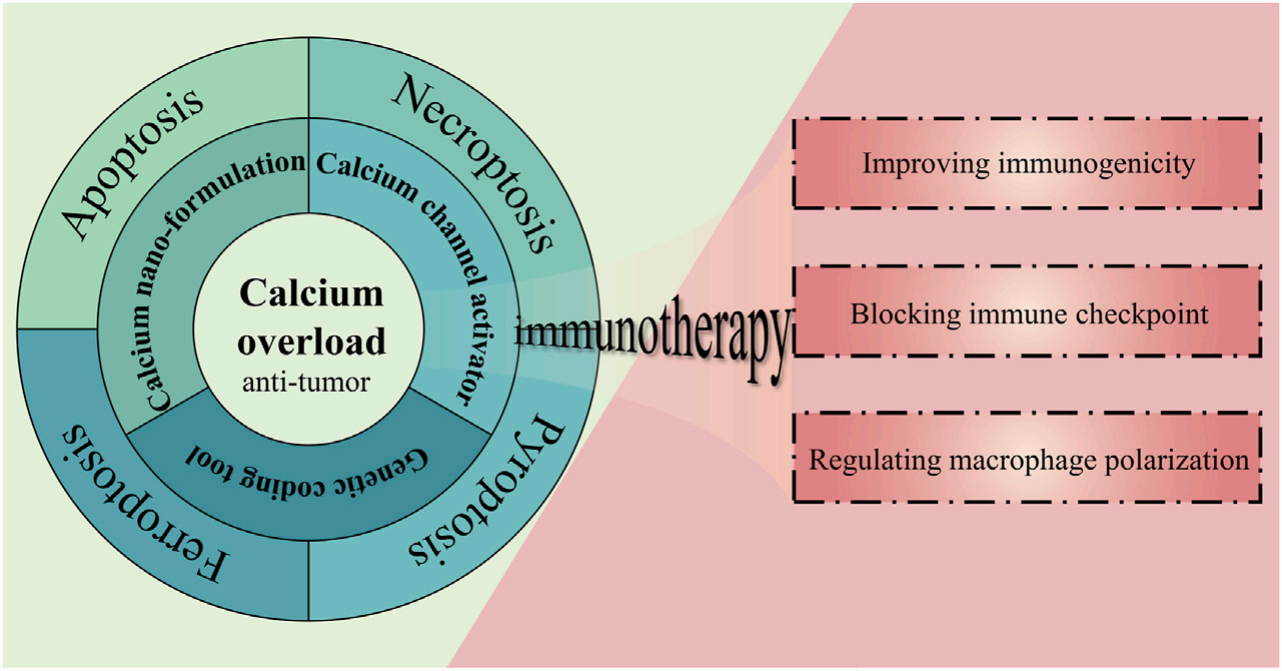
The strategies, outcomes, and prospects of calcium overload-based ion interference therapy in tumor treatment.

Therefore, calcium overload-based ion interference is considered to be a prospective anti-tumor therapy with the characteristics of disrupting calcium homeostasis in tumor cells, interfering with physiological processes related to tumor proliferation and disrupting the normal structure and function of tumor cells (Zhang et al., 2019). In addition, this therapy has also been shown to induce or augment immune responses, resulting in enhanced anti-tumor immune effects when synergistically employed with immunotherapy. (Chen et al., 2018; Zheng et al., 2021a). We have briefly described the main content of this review in Figure 1. To be specific, this review will present several strategies for implementing this therapy and summarize different outcomes of cell death mediated by this therapy, ending with a discussion of the feasibility of combining this therapy with immunotherapy.

## 2 Incentive strategies of calcium overload-based ion interference therapy

### 2.1 The conventional calcium channel activators

Currently, calcium overload-based ion interference therapy primarily relies on conventional calcium channel activators. These activators can act on various types of intracellular calcium channels and result in a significant elevation in intracellular Ca^2+^. Here, we have summarized some conventional calcium channel activators with significant anti-tumor properties in Table 1. These conventional activators can disrupt intracellular calcium homeostasis and cause calcium overload through a variety of ways.

**TABLE 1:** The conventional calcium channel activators with the ability to induce calcium overload in tumor cells.

Target	Activator	Cancer type	Cell death type	Mechanism	References
**SOCE**	Melittin	Melanoma	Apoptosis	Calcium overload disrupt mitochondrial integrity, leading to the opening of the mitochondrial permeability transition pore and ultimately to the release of apoptotic proteins	Nakagawa et al. (2020)
	DIM	Gastric cancer	Apoptosis and autophagy	DIM significantly triggered intracellular calcium overload by targeting the activation of SOCE, which in turn enhances p-AMPK/p-ACC-mediated ER stress	Ye et al. (2021)
**VGCE**	Aspirin, salicylate	Melanoma	Apoptosis and necrosis	Aspirin and salicylate can disturb calcium homeostasis, which can cause mitochondrial dysfunction and cell death by ROS-dependent depolarization and VGCE activation	Fujikawa et al. (2020)
	Diallyl trisulfide	Melanoma	Apoptosis	Diallyl trisulfide may cause mitochondrial calcium overload through VGCE leading to apoptosis	Nakagawa et al. (2020)
**MCU**	Neochlorogenic acid	Liver cancer	Apoptosis	Neochlorogenic acid can upregulate the expression of MCU to permit excess Ca^2+^ influx, which will result in mitochondrial calcium imbalance, dysfunction and structure alteration	Li et al. (2021b)
	RY10-4	Breast cancer	Apoptosis	MCU has been shown to be upregulated upon RY10-4 treatment, which can lead to mitochondrial calcium overload and disruption of mitochondrial function	Xue et al. (2021)
**TRPV**	Capsaicin	Thyroid cancer	Apoptosis	Capsaicin treatment may trigger Ca^2+^ influx by TRPV1 activation, bringing about mitochondrial calcium overload and apoptosis	Xu et al. (2020)
	Lidocaine	Glioblastomas	Pyroptosis	Lidocaine can activate CaMKII, which will phosphorylate TRPV1 and induce calcium overload in glioblastoma cells. At the same time, it will upregulate the expression of caspase-3 and GSDME proteins, thereby provoking pyroptosis	Zhou et al. (2021)
**TRPC**	Doxorubicin	Breast cancer	Apoptosis	Doxorubicin triggers persistent cytosolic Ca^2+^ release, which can stimulate the proapoptotic pathway and mitochondrial calcium overload. Meanwhile, it suppresses the pro-survival ERK1/2 pathway	Abdoul-Azize et al. (2018)
**NCX**	Bepridil	Glioblastomas and melanoma	Apoptosis	Bepridil can cause Ca^2+^-dependent cell cycle arrest by blockading the forward of NCX.	Hu et al. (2019), Liu et al. (2022c)
**NQO1**	MAM	Glioblastomas	Necrosis	MAM may trigger a non-apoptotic regulated necrosis in glioblastoma cells. By targeting NQO1, which can activate O_2_^−^/Ca^2+^/JNK1/2 pathway	Yu et al. (2020)
**TLR4**	Cucurbitacin B	Lung cancer	Pyroptosis	Cucurbitacin B can activate the NLRP3 inflammasome by directly interacting with TLR4. Besides, it can increase the mitochondrial ROS generation, Tom20 accumulation, and Ca^2+^ aggregation	Yuan et al. (2021)
**Target unknown**	δ-tocotrienol	Melanoma	Paraptosis and apoptosis	δ-tocotrienol can disruption Ca^2+^ homeostasis, with ER-derived Ca^2+^ accumulating in mitochondria and activating the paraptotic signaling. Meanwhile, it can trigger ER stress-mediated apoptosis	Montagnani Marelli et al. (2016), Raimondi et al. (2021)
	Paroxetine	Breast cancer	Apoptosis	Paroxetine can trigger an increase in intracellular and mitochondrial ROS generation and intracellular Ca^2+^ levels, so that it can induce p38-MAPK activation and begin the apoptotic process	Cho et al. (2019)
	Tilapia piscidin 4	Synovial sarcoma	Necrosis	Tilapia piscidin 4 could cause calcium overload, which can induce mitochondrial hyperpolarization, and oxidative stress in synovial sarcoma cells	Su et al. (2021)
	MHO7	Breast cancer	Apoptosis	MHO7 could trigger ER stress and apoptosis through PERK/eIF2α/ATF4/CHOP pathway	Wen et al. (2022)
	CD38	Head and neck squamous cell carcinoma	Pyroptosis	Calcium regulator CD38 could promote NLRP3 expression via Ca^2+^-NFAT signaling and subsequently induce GSDMD cleavage by activating caspase-1 activity, ultimately leading to pyroptosis	Zhang et al. (2020)

ATF4: activating transcription factor 4; ACC: acetyl CoA carboxylase; AMPK: AMP-activated protein kinase; BIM: Bcl-2, interaetion mediator of cell death; CHOP: CCAAT, enhance-binding protein homologous protein; CaMKII: Calcium-calmodulin -dependent protein kinase II; DIM: 3; 3′-Di indolyl methane; ERK: extracellular regulated kinase; ER: endoplasmic reticulum; eIF2α: eukaryotic translation initiation factor 2α; GSDME: Gasdermin E; GSDMD: Gasdermin D; IP3R1: inositol 1,4,5-triphosphate receptor 1; JNK: c-Jun N-terminal kinase; MAM: 2-methoxy-6-acetyl-7-methyljuglone; MCU: mitochondrial calcium uniporter; MAPK: microtubule-associated protein kinase; NCX: Na^+^/Ca^2+^.

Exchanger; NFAT: nuclear factor of activated T cells; NQO1: NAD(P)H: quinine oxidoreductase 1; NLRP3: NOD-like receptor thermal protein domain associated protein 3; PERK: protein kinase R-like endoplasmic reticulum kinase; ROS: reactive oxygen species; SOCE: store-operated calcium entry; TLR: toll-like receptors; TRPV: transient receptor potential vanilloid; TRPC: transient receptor potential canonical; VGCE: voltage-gated calcium entry.

The meaning of the bold values are clearer display of different types.

Firstly, with the continuous activation of the calcium channels located on the cell membrane, the intracellular Ca^2+^ accumulate abnormally so as to reach the level of calcium overload. Ye et al. found that 3, 3′-Diindolylmethane (DIM) could apparently inhibit proliferation and induce apoptosis as well as autophagy in two gastric cancer cell lines (Ye et al., 2021). Following DIM treatment, activation of stromal interaction molecule 1 (STIM1)-mediated store-operated Ca^2+^ entry (SOCE) could lead to sustained cytoplasmic calcium overload. Mechanistically, DIM activated SOCE, which apparently increase intracellular Ca^2+^ and subsequently led to the activation of p-AMPK/p-ACC expression and ER stress. Additionally, as well as promoting the inward flow of Ca^2+^, calcium overload could also be provoked by blocking the efflux of Ca^2+^. Hu et al. revealed that Bepridil could significantly elevate intracellular calcium concentrations and rapidly kill glioma cells by blocking the forward mode of Na^+^/Ca^2+^ exchanger (Hu et al., 2019). Furthermore, it has been confirmed that Bepridil wound not affect the growth of normal glial cells. In addition to affecting calcium channels on the cell membrane, certain activators can interact with calcium channels localized on organelles and release internal calcium stores. Neochlorogenic acid has been confirmed to upregulate the expression of mitochondrial calcium uniporter (MCU) which could permit excess Ca^2+^ influx (Li et al., 2021b). Analogously, when Yue et al. used the Sirtuin-1 inhibitor inauhzi to treat colorectal cancer (CRC), they demonstrated that inauhzi inhibited CRC by promoting acetylation of the MCU, which in turn enhanced mitochondrial Ca^2+^ uptake and caused mitochondria calcium overload (Sun et al., 2022). Thirdly, intracellular Ca^2+^ could be raised by indirectly affecting calcium channels. Ugur et al. found that digitalis toxin combined with microtubule-associated protein kinase (MAPK)/extracellular signal-regulated kinase inhibitors could increase intracellular calcium levels by lowering intracellular pH in certain melanoma cells. Then, it would transport extracellular Ca^2+^ into some organelles, resulting in calcium overload (Eskiocak et al., 2016).

### 2.2 Novel calcium nano-formulation

Novel calcium nano-formulations are increasingly favored by researchers in various aspects of tumor therapy for their low toxicity, excellent biocompatibility and ability to directly alter the distribution of intracellular Ca^2+^(Yao et al., 2022a; Bai et al., 2022). In Table 2, we have summarized various novel calcium nano-formulations that have displayed significant tumor-suppressive effects. For instance, Bu et al. designed and wet-chemically synthesized a class of ultra-small SH-CaO_2_ nanoparticles (Zhang et al., 2019). It was reported that transmitting the nanoparticles into tumor lesions could diminish calcium regulatory ability of the tumor and trigger a sustained cellular calcium overload effect. In this nano-system, the pH-sensitive CaO_2_ nanoparticles could slowly transform into Ca^2+^ and H_2_O_2_ in the mildly acidic tumor microenvironment (TME), concurrently triggering intracellular calcium overload and oxidative stress. However, introducing a large amount of exogenous Ca^2+^ would inevitably disrupt intracellular homeostasis and cause acute inflammatory reactions. It becomes an ideal anti-tumor strategy to mediate endogenous calcium overload *in situ* without introducing exogenous ions (Liu et al., 2020; Chu et al., 2021). Therefore, a nano-drug delivery system named UC-ZIF/BER was synthesized by Bu et al. Upon 980 nm near-infrared light stimulation, the release of nitric oxide from UC-ZIF/BER activated ryanodine receptors which rapidly increased intracellular Ca^2+^ concentration. At the same time, the calcium pump inhibitor berbamine specifically blocked Ca^2+^-ATPases on the cell membrane and thereby induced endogenous calcium overload (Chu et al., 2021). Moreover, calcium nano-formulations could combine with several efficient therapies, such as chemodynamical therapy, phototherapy, sonodynamic therapy. This strategy has shown greater anti-tumor effects and much broader application prospects. Ni et al. developed semiconductor polymer nanoparticles modified with capsaicin, which could cause intracellular calcium overload through near-infrared light stimulation without introducing additional Ca^2+^ (Schwartz et al., 2022). Specifically, under near-infrared light stimulation, it can release capsaicin to activate transient receptor potential vanilloid (TRPV1), leading to an influx of Ca^2+^ into cells. Moreover, singlet oxygen generated by nanoparticles can also induce phototherapy. Many ions such as iron, copper, and cobalt also have a significant effect on various physiological activities of cells. Therefore, the composite calcium nano-formulation based on multiple ions synergistically would theoretically exert more powerful anti-tumor ability. Yan et al. constructed a cascade nanocatalytic platform by curcumin and transferrin co-loaded CaO_2_ nanoparticles (Yin et al., 2021). This nanocatalytic platform could target to tumor and achieves highly efficient anti-tumor effects by interfering with the metabolic processes of calcium and iron ions. It was a great combination of ferroptosis, calcium overload therapy and chemotherapy.

**TABLE 2:** Novel calcium nano-formulations show great tumor-suppressive effects.

Type	Therapy	Calcium nano-formulation	Cancer type	References
**CaCO**_**3**_	Calcium overload	CaCO_3_@KAE	Lung cancer	Li et al. (2021c)
	Calcium overload, CT	^PEG^ CaNM_CUR+CDDP_	Breast cancer	Zheng et al. (2021b)
	Calcium overload, PT	CaCO_3_@COF-BODIPY-2I@GAG	Colon cancer, breast cancer	Guan et al. (2020)
	Calcium overload, IT	OVA@CaCO_3_	Colon cancer	An et al. (2020)
	Calcium overload, CT, SDT	ECaC nano-system	Colon cancer	Li et al. (2022)
	Calcium overload, CT, CDT	CM@CaCO_3_@SAF NPs@DOX	Lung cancer	Yao et al. (2022b)
	Calcium overload, CDT, SDT	Cu/CaCO_3_ @Ce6	Breast cancer	Zhao et al. (2022)
	Calcium overload, CDT, PT, IT	Cu_2_O@CaCO_3_	Colon cancer	Chang et al. (2020)
**CaP**	Calcium overload	LDM	Lung cancer	Fu et al. (2023)
	Calcium overload, CT	RGD-CaPO/DOX	Ovarian cancer	Qiu et al. (2022)
	Calcium overload, PT	GMCD	Breast cancer	Fu et al. (2021a)
	Calcium overload, SDT, IT	TiO_2_@CaP	Breast cancer	Tan et al. (2021)
	Calcium overload, CT, CDT	PGC-DOX	Breast cancer	Fu et al. (2021b)
	Calcium overload, IT, PT	MICaP	Breast cancer	Zhu et al. (2023)
**CaO₂**	Calcium overload	CaO_2_@HMSNs-PAA	Prostate cancer	Wu et al. (2020)
	Calcium overload	SH-CaO_2_ NPs	Cervical, breast, and lung carcinoma	Zhang et al. (2019)
	Calcium overload, CT	CaO_2_@ZIF-8@DOX@HA	Ovarian cancer	Sun et al. (2021)
	Calcium overload, PT	BPQD@CaO_2_ -PEG-GPC3Ab	Liver cancer	Guo et al. (2023)
	Calcium overload, CDT	CaO_2_@TA-Fe^III^	Breast cancer	Chen et al. (2021)
	Calcium overload, IT, PT	CaO_2_ @CuS-MnO_2_ @HA	Breast cancer, colon cancer	Huang et al. (2022)
	Calcium overload, IT, CDT	(Cu_2_Se-CaO_2_) @LA	Breast cancer	Feng et al. (2023)
**HAp**	Calcium overload	HAPNs	Lung cancer, osteosarcoma, melanoma	Sun et al. (2016), Wu et al. (2019), Wu et al. (2022)
	Calcium overload, CT	DOX@MSNs/HAP	Liver cancer, breast cancer	Kang et al. (2019)
	Calcium overload, PT	GA@HAP/ICG-NPs	Liver cancer	Cheng et al. (2021)
	Calcium overload, IT	nHA/GM-CSF hydrogel	Melanoma	Chen et al. (2022)

CT: chemotherapy; SDT: sonodynamic therapy; PT: phototherapy; CDT: chemodynamic therapy; IT: immunotherapy; CaCO_3_: calcium carbonate; CaP: calcium phosphate; CaO₂: calcium peroxide; HAp: hydroxyapatite.

The meaning of the bold values are clearer display of different types.

### 2.3 Calcium channels-based gene coding tools

With the great development of gene editing technology, a series of gene encoding tools represented by optogenetic tools have been designed to achieve the precise regulation of various types of physiological processes within cells. Optogenetics is pioneered by the noted scientist Karl Deisseroth. It combines optical and genetic methods to accurately manipulate diverse physiological processes in specific cells of a living tissue (Tan et al., 2022). Recently, a large number of novel calcium channel-based optogenetic tools have been developed to manipulate calcium signaling pathway and influence the expression of related genes (Tan et al., 2022). For example, He et al. presented a light-controlled calcium channel named Opto-CRAC that can respond to 470 nm blue-light irradiation (He et al., 2015). It is composed of photosensitive protein LOV2 and stromal interaction molecule (STIM) 1 fragment, which achieves precise spatiotemporal control of Ca^2+^ release-activated Ca^2+^ (CRAC) channels through light stimulation. Moreover, it has been proven that Opto-CRAC could regulate immune responses and inhibit the proliferation of melanoma in mouse model. However, the calcium signaling induced by Opto-CRAC is dependent on the expression of ORAI proteins. The further application of Opto-CRAC in cells and tissues expressing ORAI at low levels is restricted. To overcome the limitation of Opto-CRAC, a novel light-controlled calcium channel called LOCa was developed by He et al. LOCa is a highly selective calcium channel precisely regulated and activated by blue light. It enables precise optical control over Ca^2+^ signals and hallmark Ca^2+^-dependent physiological responses (He et al., 2021). When the cells were exposed to a blue light the calcium ion gates opened. When the light was turned off, the gates closed. Furthermore, LOCa has been shown to be useful in delaying Alzheimer’s disease and inhibiting abnormal self-renewal of cancerous hematopoietic stem cells. However, it is regrettable that the application of light-controlled calcium channels, including Opto-CRAC and LOCa, in the field of tumor therapy is relatively rare and further exploration is still needed. Moreover, considering that the limited penetration of light may restrain its efficacy in deep tumors, some genetic coding tools that utilize chemical factors to manipulate calcium channels have been developed. Wang et al. reported a caffeine operated synthesis module (COSMO) that regulates calcium channels through caffeine, its metabolites (Wang et al., 2021). Even more impressively, it also can be activated by caffeinated beverages, including tea, coffee and energy drinks. Furthermore, how to target these tools to tumor cells *in vivo* is an urgent problem to be solved. One way to solve this problem is to bind these tools with tumor specific promoters. At the same time, according to the location of the patient’s tumor and systemic metastasis, we can choose different delivery methods, including systemic administration, intratumoral injection, etc. Moreover, we can combine optogenetic tools with upconversion nanoplates (Yu et al., 2019). Using upconversion nanomaterials as light sensors, near-infrared light with strong tissue penetration can be converted into blue light, and then optogenetic tools can be activated *in situ*. Meanwhile, we can reduce the side effects on normal tissues as much as possible by giving targeted light or chemicals. With the rapid development of nanomedicine, it is also possible to combine these tools with suitable nanocarriers. In addition, cells can not only perceive external light and chemical signals, but also respond to mechanical stress. Therefore, mechanical genetics is regarded as a new type of induction method (Song et al., 2022). It achieves non-invasive remote control of cells by converting mechanical stress into the control of cellular genetic. Robert B. et al. found that the mechano-transduction of pulsed focused ultrasound could cause DNA damage and superoxide formation in some tumor cells by calcium overload, further forming a proinflammatory TME (Rosenblatt et al., 2021). Similarly, Yue et al. used ultrasound combined with microbubbles to effectively activate the mechanical sensitive channel Piezo1. In that way, it caused the influx of Ca^2+^, resulted in calcium overload and ultimately induced apoptosis in pancreatic cancer cells (Song et al., 2022).

## 3 Different outcomes of calcium overload-based ion interference therapy

Calcium overload, characterized by the abnormal accumulation of Ca^2+^ in cytoplasm and mitochondria, leads to varying degrees of cell damage and even cell death through various mechanisms. In this process, different types of tumors and divergent induction conditions will induce assorted types of cell death.

### 3.1 Calcium overload and apoptosis

**FIGURE 2:**
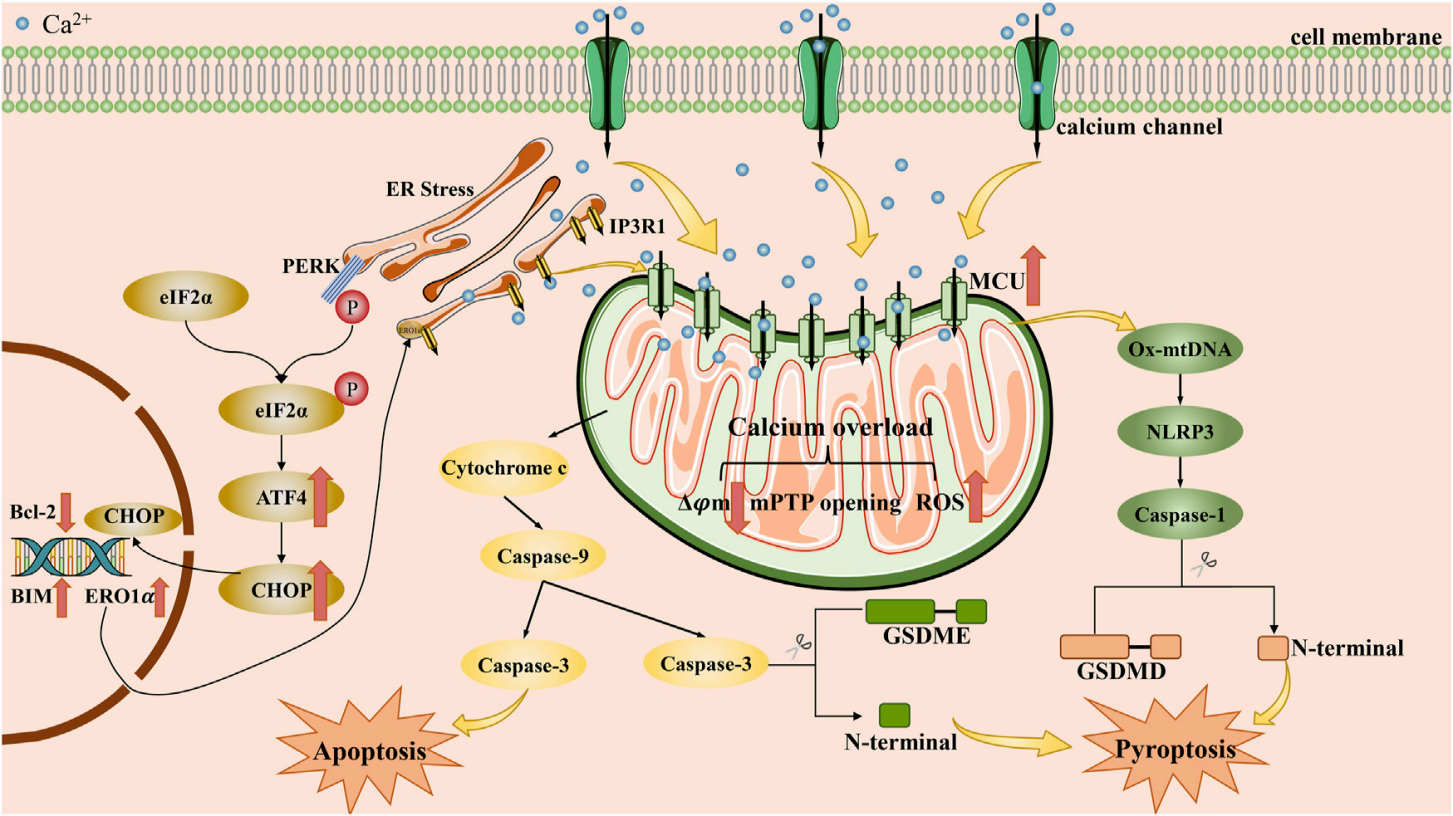
The main mechanisms by which calcium overload exerts its tumor suppressive effect by apoptosis and pyroptosis. Intracellular calcium overload, caused by the influx of exogenous calcium ions into the cytoplasm or the release of intracellular calcium stores, can induce apoptosis or pyroptosis in tumor cells, mainly through endoplasmic reticulum stress and mitochondrial dysfunction pathways. ATF4: activating transcription factor 4; BIM: Bcl-2 interaetion mediator of cell death; Bcl-2: B-cell lymphoma-2; CHOP: CCAAT enhance-binding protein homologous protein; eIF2α: eukaryotic translation initiation factor 2α; ERO1α: endoplasmic reticulum oxidoreductin-1α; ER: endoplasmic reticulum; GSDME: Gasdermin E; GSDMD: Gasdermin D; IP3R1: inositol 1,4,5-triphosphate receptor 1; MCU: mitochondrial calcium uniporter; mPTP: mitochondrial permeability transition pore; NLRP3: NOD-like receptor thermal protein domain associated protein 3; Ox-mtDNA: oxidized-mitochondrial DNA; PERK: protein kinase R-like endoplasmic reticulum kinase; ROS: reactive oxygen species.

Apoptosis is the main type of cell death that induced by calcium overload, which is closely related to mitochondrial dysfunction and ER stress. We describe the role of mitochondrial dysfunction and ER stress in Figure 2. Notably, mitochondria are the core sites of calcium overload in tumor cells (Miwa et al., 2022). Triggering Ca^2+^-dependent mitochondrial dysfunction requires the accumulation of intracellular Ca^2+^ to a certain threshold and long-term maintenance at this level (Zhang et al., 2022). This process is closely related to the MCU located on the inner membrane of mitochondria (Marchi et al., 2020). Xue et al. used RY10-4 to upregulate the expression of MCU and enhanced mitochondrial calcium uptake, thereby inducing Ca^2+^-dependent mitochondrial dysfunction and inhibiting breast cancer. When the MCU is knocked out, the mitochondrial calcium uptake induced by several stimuli is completely inhibited (Zhang et al., 2022). Mechanistically, enhanced mitochondrial calcium uptake would lead to massive mitochondrial calcium accumulation and thus promote the occurrence of mitochondrial dysfunction by affecting mitochondrial metabolism and structure (Miwa et al., 2022). However, the role of MCU in cancer cell survival appears cell line-specific or drug-specific. Xiao et al. confirmed that MCU upregulation enhanced clone formation, migration, and mitochondrial activity of endometrial cancer cells (Xiao et al., 2023). Moreover, Yu et al. indicated that MCU silencing in MDA-MB-231 cells decreased migration and invasion *in vitro* and reduced lung metastasis *in vivo* (Yu et al., 2017).

However, it is undeniable that inducing the occurrence of calcium overload in some cancer cells is indeed an effective strategy. On the one hand, mitochondrial calcium overload can have an adverse effect on mitochondrial metabolism. The elevation of Ca^2+^ increases the activity of nicotinamide adenine dinucleotide oxidase and promotes the production of reactive oxygen species (ROS) by accelerating the tricarboxylic acid cycle and oxidative phosphorylation (Cui et al., 2019). Then, varieties of ROS inhibit tumor growth through sustained cell cycle arrest (Li et al., 2021a). During this process, many key protein phosphatases was inhibited in antioxidant pathways (Perillo et al., 2020; Cheung and Vousden, 2022). However, it is notable that sublethal levels of ROS do promote tumor progression (Sies and Jones, 2020). As Jing et al. found that the mitochondrial Ca^2+^ signaling pathway mediated by the MCU regulator 1 in hepatocellular carcinoma could promote epithelial-mesenchymal transition via activating the ROS/nuclear factor erythroid 2-related factor 2 (Nrf2)/Notch1 pathway by sublethal levels of ROS(Jin et al., 2019). On the other, calcium overload can be hazardous to the structure of mitochondria. Recent decade, researches have confirmed that mitochondria calcium overload can decrease mitochondrial membrane potential and open the mitochondrial permeability transition pore (MPTP), which will result in mitochondrial swelling and the rupture of the outer mitochondrial membrane (Zhang et al., 2022).

ER stress is another significant mechanism implicated in apoptosis triggered by calcium overload. The ER regulates the influx and efflux of Ca^2+^ mainly through ion channels on its membrane such as ryanodine receptor (RyR), inositol 1,4,5-triphosphate receptor (IP_3_R) and sarcoendoplasmic reticulum calcium transport ATPase (SERCA), ensuring proper calcium storage and adequate folding capacity (Zheng et al., 2022b). Once the calcium regulation mechanism of ER is disturbed, the ER calcium homeostasis would collapse, resulting in severe ER stress and crosstalk with mitochondrial dysfunction. According to Zhang et al., curcumin induced ER stress to inhibit thyroid cancer cells, while simultaneously initiating the mitochondrial apoptotic pathway by inhibiting the activity of SERCA2 and activating the calcium/calmodulin-dependent protein kinase (CaMK) II/c-Jun N-terminal kinase (JNK) pathway (Zhang et al., 2018). In parallel, the unfolded protein response (UPR) is also activated in reply to ER stress. Although the UPR might help reduce the load of unfolded proteins to maintain cell survival (Marciniak et al., 2022), persistent UPR caused by long-term severe ER stress could induce apoptosis through protein kinase R-like endoplasmic reticulum kinase (PERK)/eukaryotic translation initiation factor 2α (eIF2α)/activating transcription factor 4 (ATF4)/CCAAT enhance-binding protein homologous protein (CHOP) pathway (Hetz, 2012; Yu et al., 2021b; Coker-Gurkan et al., 2021). As a transcription factor, CHOP promotes mitochondrial dependent apoptosis by upregulating the expression of pro-apoptotic proteins comprise BAX, BAK and BIM. Meanwhile, it can downregulate the expression of anti-apoptotic proteins contain BCL-2, BCL-XL and MCL-1 (Zhou et al., 2022). Moreover, CHOP can also contribute to the death receptor pathway. For instance, CHOP can upregulate the expression of death receptor (DR) 4 or DR5, so that it can sensitize a variety of cancer cells to trail mediated apoptosis (Hu et al., 2018; Kim et al., 2019; Cao et al., 2020). In addition, Glutamine-rich protein 1 (QRICH1) has also been revealed as a key effector of the PERK/eIF2α/ATF4/CHOP pathway. QRICH1 controls proteostasis in ER by regulating protein translation and secretion, thereby determining cell fate at the end of the UPR(You et al., 2021). Recently, Recently, a research team from the University of Geneva unveiled a direct crosstalk between SOCE and UPR via inositol-requiring enzyme 1(IRE1), acting as key regulator of ER Ca^2+^ and proteostasis in T cells (Carreras-Sureda et al., 2023). Under ER stress, the IRE1-STIM1 axis may boost SOCE to preserve immune cell function. Maybe, the IRE1-STIM1 axis could be a significant pathway for cancer immunotherapy.

Additionally, there is a close contact between the mitochondrial membrane and the ER membrane, known as the mitochondrial associated endoplasmic reticulum membrane (MAM) (Loncke et al., 2021). One of the main functions of MAM is to mediate the flow of calcium ions between ER and mitochondria. Increasing Ca^2+^ transfer from ER to mitochondria is also an effective means of inducing calcium overload (Wu et al., 2023). Xie et al. confirmed that TAT-fused inositol 1,4,5-trisphosphate receptor-derived peptide targeting the BH4 domain of Bcl-2 increased cisplatin-induced Ca^2+^ flux from ER to mitochondria, thereby promoting apoptosis and enhancing cisplatin cytotoxicity in ovarian cancer cells (Xie et al., 2018). However, a large amount of research is still needed to confirm the targeted key factors in MAM. The cell line-specific and drug-specific also need to be explored.

### 3.2 Calcium overload and pyroptosis

Pyroptosis is a type of programmed cell death characterized by an inflammatory response, which is facilitated by the gasdermin family of proteins that disrupt the cell membrane. Its primary characteristics include cell swelling and the release of interleukin-1β. (Yu et al., 2021a). As research continues to deepen, more and more mechanisms of pyroptosis continue to be discovered, and at present, two main pathways and several alternative pathways have been recognized (Loveless et al., 2021). Among the classical pathways, pyroptosis is mainly mediated by gasdermin D and involves cysteinyl aspartate specific proteinase (caspase)-1 or caspase-4/5/11. One alternative pathway that has garnered significant attention is the caspase-3/gasdermin E pathway. Moreover, Ca^2+^ was found to regulate pyroptosis mainly by affecting activators of gasdermin proteins, including NOD-like receptor thermal protein domain associated protein 3 (NLRP3) inflammasome, caspase-3. Here, we describe the we describe the main mechanisms by which calcium overload leads to pyroptosis in Figure 2. Earlier studies have demonstrated that Ca^2+^ accumulation is tightly associated with the assembly and activation of NLRP3 inflammasome. It means that blocking intracellular calcium accumulation will arrest inflammasome activation (Murakami et al., 2012; Akbal et al., 2022). Geun Shik Lee et al. confirmed that hindering the release of Ca^2+^ from calcium stores will interrupt the activation of NLRP3 inflammasome (Lee et al., 2012). Subsequently, the activated NLRP3 inflammasome facilitates pyroptosis by promoting the cleavage of GSDMD protein through caspase-1. Additionally, activated NLRP3 inflammasome can accelerate Ca2+ accumulation, leading to the formation of positive feedback.

Pyroptosis can lead to membrane rupture, which is manifested by the formation of pores on the membrane the appearance of small pores on the cell membrane, so as to favor the influx of Ca^2+^. Yet NLRP3 alone can also promote the efflux of Ca^2+^ from the ER by regulating RyR2 and increase intracellular calcium levels (Kong et al., 2021). Yuan et al. demonstrated that during the process of cucurbitacin B inhibited non-small cell lung cancer, the release of Ca^2+^ from intracellular calcium stores and the occurrence of mitochondrial calcium overload are important for the toll-like receptors 4/NLRP3/gasdermin D pathway (Yuan et al., 2021). However, the specific mechanism by which accumulated Ca^2+^ promotes NLRP3 inflammasome activation requires further investigations. Nowadays, two possible molecular mechanisms have been identified: (1) Excessive Ca^2+^ transport from cytosol to mitochondria causes mitochondrial dysfunction. It would drive the release of oxidized-mitochondrial DNA into cytosol via MPTP and voltage-dependent anion channel, which has been proven that it is profit to the activation of NLRP3 inflammasome (Moossavi et al., 2018; Xian et al., 2022); (2) Ca^2+^ could directly contribute to the activation of NLRP3 inflammasome by promoting NLRP3 interaction with apoptosis-associated speck-like protein containing a CARD (Kong et al., 2021). More investigations are needed to be designed and further reveal the underlying mechanism by which Ca^2+^ promote the activation of NLRP3 inflammasome.

Beside regulating major pathways, Ca^2+^ also takes part in alternative pathways, particularly the caspase-3/gasdermin E pathway. Calcium overload could accelerate the assembly of a pyroptosome complex consisting of Apaf-1 and caspase-4 by inducing mitochondrial permeability transition. After the activation of a pyroptosome complex, caspase-3 and gasdermin E would be cleaved, thus activate gasdermin E-mediated pyroptosis (Xu et al., 2021). Similarly, a novel calcium nano-modulator constructed by Zhen et al. induced the occurrence of mitochondrial calcium overload with multiple effects, including GSDME-mediated pyroptosis (Zheng et al., 2022a).

### 3.3 Calcium overload and necroptosis

**FIGURE 3:**
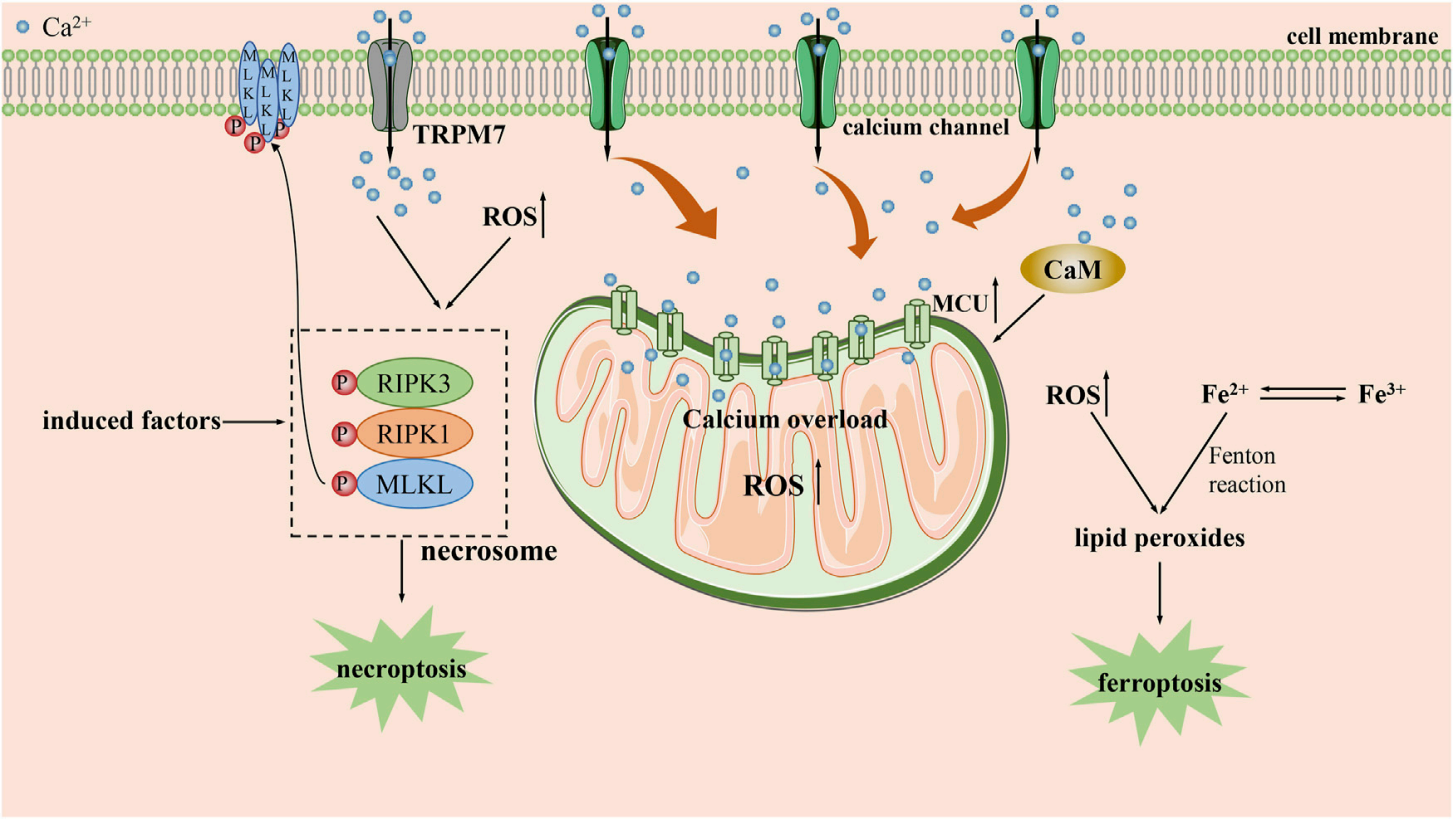
The main mechanisms by which calcium overload exerts its tumor suppressive effect by necroptosis and ferroptpsis. CaM: calcium/calmodulin-dependent protein; MCU: mitochondrial calcium uniporter; RIPK1: receptor interacting serine/threonine protein kinase 1; RIPK3: receptor interacting serine/threonine protein kinase 3; ROS: reactive oxygen species; TRPM7: transient receptor potential melastatin 7.

Necroptosis is a form of necrotic cell death mainly mediated by necrosome. The necrosome is a protein complex composed of receptor interacting serine/threonine protein (RIP) kinase 1, RIP kinase 3 and mixed lineage kinase region like protein (MLKL) (Yan et al., 2022). Calcium overload is recognized as be one of the key initiators of necroptosis (Faizan and Ahmad, 2021). The formation of necrosome can induce necroptosis by hastening the influx of Ca^2+^ to form calcium overload (Figure 3). An experiment on human colon cancer demonstrated that the formation of necrosome was followed by translocation to the plasma membrane (Cai et al., 2014). After that, it can facilitate a robust influx of Ca^2+^ mediated by transient receptor potential melastatin 7. Such an influx of Ca^2+^ may have a positive feedback effect and bring about the subsequent occurrence of necroptosis. Furthermore, it is reported that a large proportion of necrosome can also be found to be associated with ER and lead to ER stress (Liang et al., 2021).

Moreover, calcium overload also contributes to the formation of necrosome. While using the antimicrobial peptide PFR to treat acute myeloid leukemia (AML), Yu et al. proved that PFR triggered increased mitochondrial ROS and thus accelerated the formation of necrosome through cytoplasmic calcium overload (Lv et al., 2019). Similarly, upon the treatment of neuroblastoma with HVJ-e, it was confirmed that continuously calcium overload in the cell lead to necrosome formation by activating CaMKⅡ-mediated phosphorylation of RIP1(Nomura et al., 2014). Additionally, calcium overload can also regulate necroptosis through alternative pathways. Poly (ADP-ribose) polymerase-1(PARP1) and AIF have been shown to be involved in calcium overload mediated necroptosis (Cabon et al., 2012). Similarly, Paul et al. authenticated that prolonged fumonisin B1 exposure in neuroblastoma can induce necroptosis by calcium overload (Paul et al., 2021). During this process, the accumulation of Ca^2+^ contributes to the induction of PARP1 activation, which results in an inhibitory effect via PARP1/JNK/AIF signaling pathway to induce necroptosis.

Ca^2+^ is also involved in regulating non canonical necroptosis. During the treatment of pancreatic cancer with adipoRon, Miho Akimoto et al. demonstrated that when adipoRon induced Ca^2+^-dependent mitochondrial dysfunction through calcium overload accompanying with the activation of RIP kinase 1 and extracellular signal regulated kinase 1/2, which led to non-canonical necroptosis (Akimoto et al., 2018).

### 3.4 Calcium overload and ferroptosis

Ferroptosis is an iron-dependent cell death characterized by the accumulation of lipid peroxides (Dixon et al., 2012; Stockwell et al., 2017). Surprisingly, previous studies have shown that there is crosstalk between iron and Ca^2+^ through ROS during ferroptosis. Large amounts of ROS generated by iron can act on the ER and stimulate calcium release from calcium stores, arousing a sustained increase in intracellular Ca^2+^ by activating IP_3_R, RyR and reducing the activity of SERCA, PMCA (Núñez and Hidalgo, 2019). The persistently increased Ca^2+^ that are transported into mitochondria will subsequently stimulate lipid peroxidation, contributing to a vicious cycle that exacerbates ferroptosis (Gleitze et al., 2021). However, the role of Ca^2+^ in ferroptosis is currently still contentious.

Some studies have argued that increased Ca^2+^ may have roles in delaying or inhibiting ferroptosis. Wang et al. discovered that CaMK kinase 2 activated by Ca^2+^ in melanoma could inhibit ferroptosis by strengthening the expression of anti-oxidant proteins such as heme oxygenase 1 and superoxide dismutase 2, which involved in Nrf2-dependent anti-oxidant mechanism (Wang et al., 2022). Similarly, Bing et al. demonstrated that the leakage of Ca^2+^ from the ER would be facilitated by enhancing the interaction of cell migration-inducing protein with IP_3_R in prostate cancer cells. Afterwards, Ca^2+^ could activate CaMKII, which further accelerates Nrf2 phosphorylation and nuclear localization, contributing to the uptake of cystine which counteract ferroptosis (Liu et al., 2022a). On the contrary, some studies also suggest that Ca^2+^ may also amplify the ferroptosis process (Figure 3). For example, Peng et al. used erastin to induce calcium-dependent ferroptosis in lung cancer cells and demonstrated that Ca^2+^/CaM signaling is a key mediator of ferroptosis mediated by erasin. Blocking this signaling would significantly rescue cell death by inhibiting ferroptosis (Chen et al., 2020). Analogously, some research have demonstrated that MCU-dependent mitochondrial calcium overload is integral for the induction of ferroptosis by long-term cold stimulation (Nakamura et al., 2021).

A recent study on ferroptosis could partly explain the contradictory influences of Ca^2+^(Pedrera et al., 2021). Early cytosolic Ca^2+^ increases could induce the activation of the endosomal sorting complex required for transport III-dependent membrane repair machinery and suspend ferroptosis. However, the progression of lipid peroxidation at the plasma membrane would not be interrupted. Furthermore, the paradoxical role of Ca^2+^ in ferroptosis may also be related to cell line heterogeneity and different stimuli.

## 4 The prospect of combining calcium overload-based ion interference therapy with immunotherapy

The discovery of immunotherapy has led to a better comprehension of tumors and significant advancements in tumor therapy. The regulation of intracellular Ca^2+^ is an indispensable part in various processes, including immune cell activation, the switching of phenotypes and TME enhancement (Vig and Kinet, 2009). For example, targeting elevated intracellular Ca^2+^ can effectively stimulate the proliferation of cytotoxic lymphocytes, ultimately enhancing their cytotoxic effect on tumor cells (Kim et al., 2017). What’s more, the activation of T lymphocyte-associated transcription factors such as nuclear factor of activated T cells, NF-κB, and JNK relies significantly on elevated levels of intracellular Ca^2+^(Trebak and Kinet, 2019; Meng et al., 2020). Consequently, the integration of calcium overload-based ion interference therapy with immunotherapy has the potential to substantially enhance the antitumor effect by regulating Ca^2+^. This is particularly true with the advancement of nanomedicine, which may propel the application of this therapy.

Lack of immunogenicity and insufficient activation of antitumor immune responses are key reasons contributing to the insensitivity of certain tumors to immunotherapy (Huang et al., 2021). Based on this, targeting improved immunogenicity to promote immunogenic cell death (ICD) has become a key aspect in immunotherapy. In this process, the role of multiple damage associated molecular patterns (DAMPs), including high mobility group box-1, calreticulin (CRT) and heat shock proteins, cannot be ignored (Galluzzi et al., 2017). Calcium overload has been confirmed to significantly promote the release of DAMPs to improve immunogenicity and efficiently enhance the anti-tumor immune effect (Zheng et al., 2021a). Zheng et al. developed a multifunctional calcium nano-formulation named ^PEG^CaCUR, which significantly enhanced the release of DAMPs by inducing mitochondrial calcium overload. Subsequently, a significantly higher proportion of mature dendritic cells and activated T cells was observed, confirming the successful ICD elicitation (Zheng et al., 2021a). Furthermore, ER stress induced by calcium overload also contributes to the higher immunogenicity. The release of Ca^2+^ from the calcium store may also facilitate the exposure of CRT localized in the ER, contributing to better improve immunogenicity (Tufi et al., 2008). CRT exposure on the cell surface is known to deliver robust pro-phagocytic signals to myeloid cells (Guilbaud et al., 2023) For example, surface-exposed CRT has been shown to be fundamental for the uptake of dying cancer cells by dendritic cell precursors that can initiate adaptive tumor-targeting immune responses (Obeid et al., 2007). Dai et al. reported a novel ICD nanoinductor that enhanced CRT exposure by facilitating the release of Ca^2+^ from the store into the cytosol, which eventually triggered ICD(Dai et al., 2020). What’s more, severe ER stress in conjuction with oxidative stress will result in the release and exposure of more DAMPs.

With the exception of improving immunogenicity, targeting tumor associated macrophages (TAMs) is also an effective immunotherapy. TAMs mainly consist of M1 phenotype associated with anti-tumor activity and M2 phenotype associated with pro-tumor activity (Christofides et al., 2022). There are three strategies which can target TAMs to improve the TME: (1) directly clearing M2 type macrophages; (2) accurately inhibiting the recruitment of macrophages; (3) reprograming TAMs from M2 to M1 phenotype (Xiang et al., 2021). Numerous research have shown that the concentration of Ca^2+^ in TAMs might be closely related to the phenotype of TAM(Chen et al., 2018; Kang et al., 2018; Bi et al., 2020). For example, Chen et al. successfully switched TAMs from tumor-promoting M2 phenotype to tumor-inhibiting M1 phenotype with chloroquine (Chen et al., 2018). Mechanically, chloroquine can directly act on lysosomes, releasing Ca^2+^ stored in lysosomes. For one thing the increased cytoplasmic Ca^2+^ can activate p38 and NF-κB for polarizing TAMs to M1 phenotype, for another it can stimulate transcription factor EB for reprograming the metabolism of TAMs. Similarly, the use of near-infrared light to assist in regulating intracellular calcium levels also promotes macrophage polarization towards M1 phenotype by increasing intracellular Ca^2+^(Kang et al., 2018). Furthermore, ROS and nicotinamide adenine dinucleotide phosphate have also been considered as crucial players in the regulation of macrophage polarization (Mehla and Singh, 2019). During the suppression of AML progression by chenodeoxycholic acid, chenodeoxycholic acid could induce mitochondrial dysfunction via calcium overload and subsequently promote lipid peroxidation via the ROS/p38-MAPK/Diacylglycerol-O-Acyltransferase 1 pathway (Liu et al., 2022b). Besides, chenodeoxycholic acid has also been shown to inhibit M2 macrophage polarization, but the specific mechanism is unknown. Certainly, it can be speculated that the massive ROS and lipid peroxidation induced by calcium overload play a critical role in this process. However, further studies will be necessary for us to verify and reveal the specific mechanisms.

Immuno-checkpoint blocking therapy (ICB) has demonstrated great clinical application potential, but its clinical application response rate is often hindered by the tumor immune suppression microenvironment. Previous research have found that calcium signaling is bound up with the efficacy of ICB(Freitas et al., 2018). A nano-formulation named CaNP@CAD-PEG, constructed by An et al. not only takes advantages of the calcium overload to enhance immunogenicity and promote TAM reprogramming, but also achieves tumor cell specific programmed cell death ligand 1 silencing via a calcium activated DNAzyme, significantly boosting the antitumor immune effect (An et al., 2022). Apart from directly inhibiting the primary tumor by inducing the calcium overload, the nano-sonosensitizer called TiO2@CaP, activated by the acidic TME, could further combine with anti-programmed death 1 and bring about the inhibition of growth of distal tumors and lung metastasis of tumors (Tan et al., 2021).

However, it cannot be ignored that there are certain risks in combining calcium overload-based ion interference therapy with immunotherapy. It has been shown that cytotoxic T Lymphocytes have a bell-shaped Ca^2+^ dependence with an optimum for cancer cell elimination at rather low extracellular Ca^2+^ concentrations (23–625 μm) (Zhou et al., 2018). A decrease of extracellular Ca^2+^ concentrations or partial inhibition of ORAI1 activity by selective blockers in the TME could obviously inhibit cancer growth by simultaneously increasing cytotoxic T Lymphocytes and natural killer cell cytotoxicity and decreasing cancer cell proliferation. Therefore, the influx of Ca^2+^ has at least a dual role in the TME. We believe that the most important thing is to go for a balance that maximizes the elimination of tumors while protecting the stability of one’s immune system.

## 5 Conclusion

Calcium overload-based ion interference therapy caused various cellular damages by artificially inducing the occurrence of calcium overload and triggered different types of cell death through various mechanisms to suppress the proliferation and metastasis of tumor cells. This therapy has shown momentous tumor inhibitory effects and received increasing attention. However, there are still several issues that need to be addressed:

1) Although exciting results were obtained in some clinical trials, some calcium channel activators were also found to be extremely prone to trigger strong systemic toxic side effects, or need to be applied with doses higher than the current clinically safe dosage. At the same time, while utilizing novel calcium nano-formulations can localize calcium overload to tumor sites, further clinical research is needed to investigate the safety and efficacy of these novel calcium nano-formulations.
2) While calcium overload can inhibit tumors by inducing severe cell damage, it is nonnegligible that the increased Ca^2+^ could enhance tumor proliferation and decrease apoptosis before reaching the calcium overload level. Achieving a balance between cell death and proliferation is crucial to maximize the tumor-suppressive effects of calcium overload.
3) Recently, more and more ion interference therapy based on different metal ions have been widely developed and have shown great anti-tumor effects. Theoretically, combining multiple ion interference therapy would show more efficient anti-tumor effects. Therefore, further studies are needed to explore its influence on various normal tissues and organs throughout the body in order to ensure its biosafety.
4) Due to the heterogeneity between individuals and different kinds of tumors, a “one-size-fits-all” approach is not feasible. Developing individualized and precise treatment strategies is essential to optimize therapeutic outcomes.

In conclusion, the calcium overload-based ion interference therapy has a promising clinical application for its robust antitumor effect. As our understanding of the relationship between Ca^2+^ and tumors deepens, more effective therapies and technologies are expected to emerge, along with a better understanding of the mechanisms underlying this therapy to enhance therapeutic efficacy and facilitate clinical translation.
